# Usefulness of kidney slices for functional analysis of apical reabsorptive transporters

**DOI:** 10.1038/s41598-017-12828-z

**Published:** 2017-10-09

**Authors:** Hiroshi Arakawa, Ikumi Washio, Natsumi Matsuoka, Hikaru Kubo, Angelina Yukiko Staub, Noritaka Nakamichi, Naoki Ishiguro, Yukio Kato, Takeo Nakanishi, Ikumi Tamai

**Affiliations:** 1 Department of Membrane Transport of Biopharmaceutics, Faculty of Pharmaceutical Sciences, Institute of Medical, Pharmaceutical and Health Sciences, Kanazawa University, Kakuma-machi, Kanazawa, 920-1192 Japan; 2 Pharmacokinetics and Non-Clinical Safety Department, Nippon Boehringer Ingelheim Co., Ltd, Kobe, Japan; 3 Laboratory of Molecular Pharmacotherapeutics, Faculty of Pharmaceutical Sciences, Institute of Medical, Pharmaceutical and Health Sciences, Kanazawa University, Kakuma-machi, Kanazawa, 920-1192 Japan

## Abstract

Kidney plays a key role in the elimination and reabsorption of drugs and nutrients, however *in vitro* methods to evaluate renal disposition are limited. In the present study, we investigated usefulness of isolated kidney slice, which had been used for transport only at basolateral membrane of tubular epithelial cells, for evaluation of apical membrane transporters. As transporters that are easy to discriminate between apical and basolateral transports, apical membrane specific and sodium-dependent transporters (SGLTs and OCTNs) and pH-dependent transporters (PEPTs) are selected. Uptake of ergothioneine, carnitine and methyl-α-D-glucopyranoside, which are substrates of apical Octn1, Octn2, and Sglt1/2, respectively, by mice kidney slices showed clear Na^+^ dependence and reduction by selective inhibitors. In addition, sodium dependence of ergothioneine uptake was negligible in the kidney slice from Octn1-gene deficient mice. Moreover, uptake of PepT1/2 substrate glycyl-sarcosine, was higher than that in the presence of glycyl-leucine, a non-specific Pept inhibitor. The *K*_*m*_ and *IC*_50_ values for substrates and inhibitors of each transporter were mostly comparable to those obtained in transporter-transfected cells. In conclusion, it was demonstrated that kidney slices are promising tool to study transporters expressed at the apical membranes as well as basolateral membranes of kidney tubular epithelial cells.

## Introduction

Renal handling of drugs and nutrients consists of glomerular filtration, tubular secretion and reabsorption processes and transporters often contribute to the secretion and reabsorption processes. For examples, organic anion transporters (OAT1 and OAT3) and organic cation transporter 2 (OCT2) are responsible for the uptake of drugs from blood across basolateral membranes of renal tubular cells^1^. At the apical membrane of the cells, there are various influx and efflux transporters such as sodium-dependent glucose transporters (SGLT1 and SGLT2) and pH-dependent peptide transporters (PEPT1 and PEPT2) for nutrient reabsorption and ATP-dependent multidrug resistance protein 1 (MDR1/P-gp), breast cancer resistant protein (BCRP) and multidrug-associated proteins (MRP2 and MRP4)^1–4^ for urinary excretion of xenobiotics and metabolic end products.

To date, membrane transport of various substances across the apical membranes of the renal tubular cells has been examined using cancer and artificially immortalized cultured cell models, such as human renal HK-2, HKC and Caki-1 cells^5–8^. In addition, although primary culture of human kidney tubular epithelial cells was suggested to be useful, their availability is limited^9^. We have also demonstrated that primary culture of rat proximal tubular cells is useful to evaluate apical transporters^10^. However, the primary cultured cells exhibited substantial changes of transporter expression profiles during culture, which is difficult to control and those cultured cells are technically not easy to prepare routinely. Therefore, any *in vitro* model with easier preparation method and can maintain the comparable expression of transporters as intact tissues is desirable. Kidney tissue slices have been frequently used for pharmacokinetic evaluations of basolateral transport of drugs across the tubular epithelial cells^11–14^. However, this model has not been applied to the analysis of apical membrane transport, since it has been empirically considered as appeared in the report by Wedeen *et al*. that transports at apical membrane were not assessed by kidney slices based on the unsuccessful result of autoradiographs of alpha-aminoisobutyric acid, an apically-expressed amino acid transporter substrate^15^. However, no further studies on the usefulness of kidney slices for the evaluation of the apical membrane transport have been reported, despite that it is technically simple without cultivation and considered that both of apical and basolateral membrane should contact to surrounding transport assay medium similarly. Furthermore, recent improved device technologies enable us to make thinner and uniform kidney slices (0.3 mm) compared with those by handmade. Accordingly, we expected that apical uptake transporters should be evaluated by kidney slices. Since many compounds could be substrates of transporters both at apical and basolateral membrane transporters, we selected transporters and their substrates characteristic to apical membranes; sodium-dependent transporters (SGLTs and OCTNs) and pH-dependent transporters (PEPTs). SGLT1 and SGLT2 are expressed at the apical membrane and transport D-glucose in a sodium-dependent manner which is distinct from basolateral sodium-independent GLUT transporters. We have shown that ergothioneine and L-carnitine are reabsorbed via organic cation/carnitine transporter OCTN1 and OCTN2 in a sodium dependent manner^16–20^, and transporters with similar characteristics have not been identified at the basolateral membranes. Furthermore, oligopeptide transporters PEPT1 and PEPT2 are expressed only at apical membranes. By focusing on these transporters, we could clearly detected the transport characteristics of each transporter substrate in mice kidney slice, including sodium or pH dependence, inhibitor selectivity, defect of transporter activity in OCTN1-gene deficient mice and comparable *K*_*m*_ or *IC*_50_ with those observed in each transported-overexpressed cultured cells.

## Results

### Uptake of Specific Substrates for Octn1, Octn2, Sglt1/2 and Pept1/2 by Mice Kidney Slices

**Figure 1:**
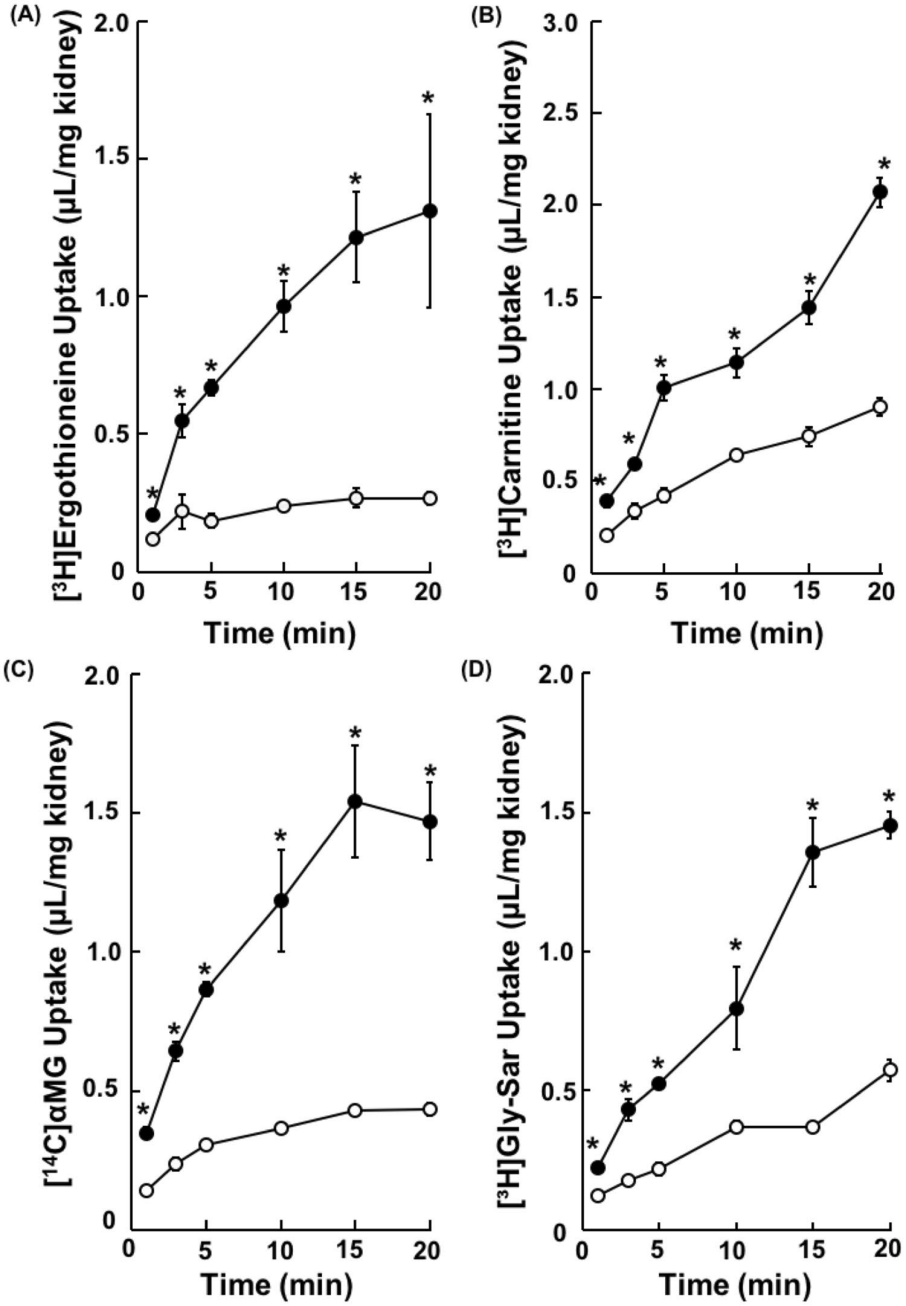
Carrier-mediated Uptake of [^3^H]Ergothioneine, [^3^H]Carnitine, [^14^C]αMG and [^3^H]Gly-Sar by Mice Kidney Slices. Uptake of (**A**) [^3^H]ergothioneine (1.0 μM), (**B**) [^3^H]carnitine (1.3 nM) and (**C**) [^14^C]αMG (2.5 μM) was performed in the presence or in the absence of Na^+^ into mice kidney slices at pH 7.4 and 37 °C for 1, 3, 5, 10, 15 and 20 min. Closed and open circles represent each substrate uptake in the presence or the absence of Na^+^, respectively. (**D**) [^3^H]Gly-Sar (36 nM) was performed in the presence or in the absence of Gly-Leu (5 mM) into mice kidney slices at pH 7.4 and 37 °C for 1, 3, 5, 10, 15 and 20 min. Closed and open circles represent [^3^H]Gly-Sar uptake in the presence or the absence of Gly-Leu, respectively. Each result represents the mean ± S.E.M (n = 3).* Indicates a significant difference from the uptake in the absence of Na^+^ or Gly-Leu at each time point (p < 0.05) by Student’s t-test.

Since Octn1, Octn2 and Sglt1/2 transport ergothioneine, carnitine and methyl-α-D-glucopyranoside (αMG), respectively, in a Na^+^-dependent manner^17,21–25^, uptake of the these three substrates into mouse kidney slices in the presence and the absence of Na^+^ was examined. Uptakes of all of [^3^H]ergothioneine, [^3^H]carnitine and [^14^C]αMG by the kidney slices were increased with time and those in the presence of Na^+^ were significantly higher than those in the absence of Na^+^ (Fig. 1A–C). Moreover, uptake of [^3^H]glycylsarcosine (Gly-Sar) was decreased in the presence of 5 mM Gly-Leu, a non-specific Pept1/2 inhibitor^26^ (Fig. 1D). Since the uptakes of all compounds by mice kidney slices was linearly increased up to 15 min, following studies were conducted by measuring uptake within 15 min as the initial influx rate. Furthermore, viability of kidney slices in this model was investigated. Uptakes of [^3^H]carnitine, [^14^C]αMG and [^3^H]Gly-Sar were maintained over 6 hrs. On the other hand, uptake of [^3^H]ergothioneine reduced to 69.0 ± 6.6% in 1 hr after removal of kidney from mice compared with those in 25 min (Supplemental Fig. 1A–D). ATP content in kidney slices in the presence and absence of Na^+^ were reduced to 61.3 ± 4.8% and 59.4 ± 7.2%, respectively, during incubation for 6 hrs (Supplemental Fig. 1E and F), showing no significant difference between in the presence and absence of Na^+^ (Supplemental Fig. 1F). Accordingly, although it is dependent on substrates or transporters, kidney slices maintain transport activity at least 1 hr or longer. Therefore, following studies were conducted within 1 hr incubation at 37^o^C.

### Effect of Octn1 Knockout on Uptake of [^3^H]Ergothioneine by Mice Kidney Slices

**Figure 2:**
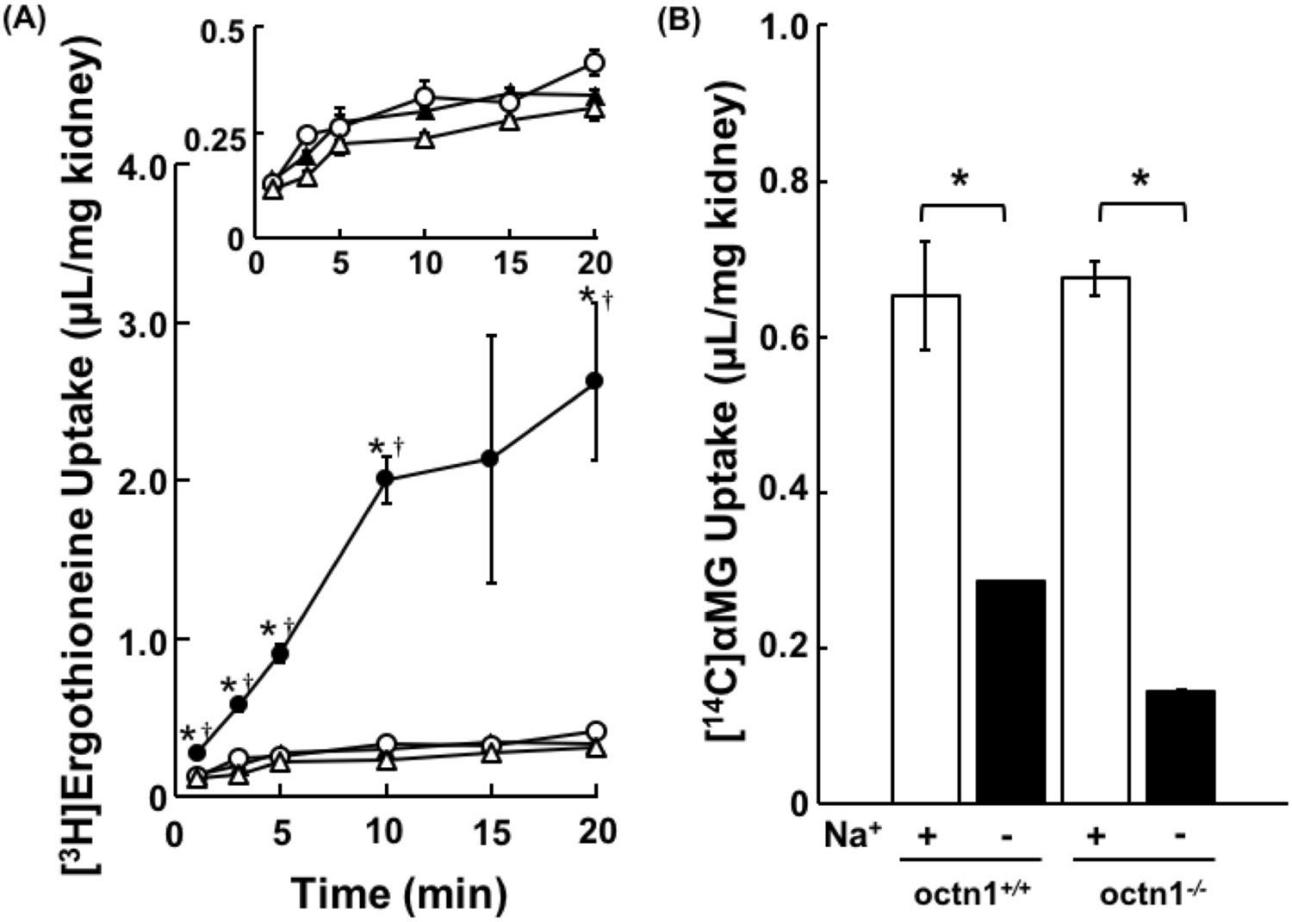
Uptake of [^3^H]Ergothioneine by Octn1^−/−^ and Wild-type Mice Kidney Slices. The uptake of [^3^H]ergothioneine (**A**) and [^14^C] αMG (**B**) into Octn1^−/−^ (open symbol) and wild-type (closed symbol) mice kidney slices. (**A**) Uptake of [^3^H]ergothioneine into kidney slices was performed at pH 7.4 and 37 °C for 1, 3, 5, 10, 15 and 20 min in the presence (circle) or the absence (triangle) of Na^+^. (**B**) The uptake of [^14^C]αMG into kidney slices was performed at pH 7.4 and 37 °C for 5 min in the presence (open bars) or absence (closed bars) of Na^+^. Each result represents the mean ± S.E.M. (n = 3 or 4). * and ^†^ indicate a significant difference from the uptake in the absence of Na^+^ (p < 0.05) or Octn1^−/−^ mice at each time point by Student’s t-test.

To clarify whether Na^+^-dependent uptake of ergothioneine was ascribed to Octn1, [^3^H]ergothioneine uptake by mice kidney slices derived from Octn1 deficient Octn1^−/−^ mice was measured (Fig. 2A). The initial uptake of [^3^H]ergothioneine was significantly reduced, and Na^+^-dependent uptake of [^3^H]ergothioneine by kidney slices was negligible in Octn1^−/−^ mice, whereas, Na^+^-dependent uptake of [^14^C]αMG by kidney slices was observed both in Octn1^−/−^ and wild-type mice (Fig. 2B). It is considered that the decreased uptake of ergothioneine by the kidney slices from Octn1^−/−^ mice is specific to Octn1.

### Characterization of Uptake Transporters by Mice Kidney Slices and Transporter-expressing Cells

**Figure 3:**
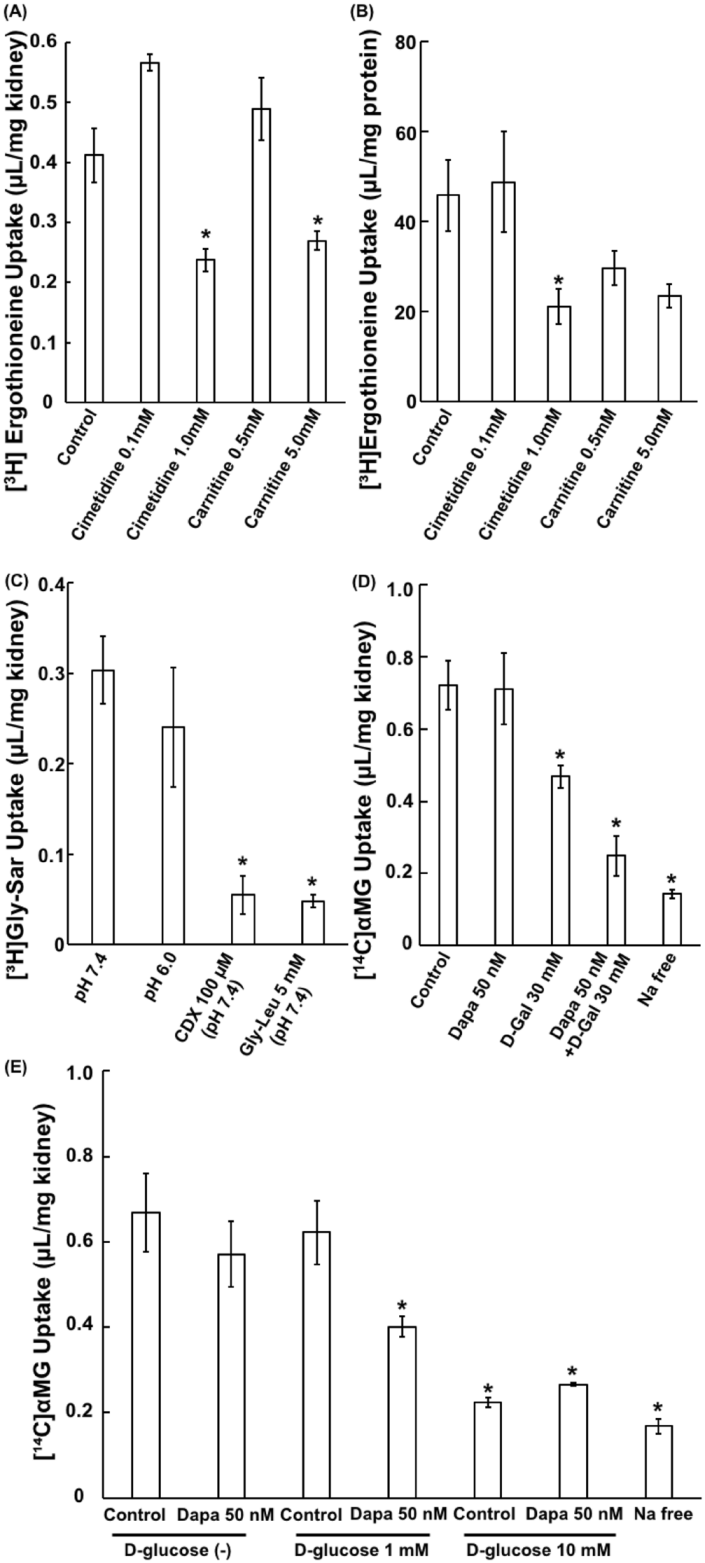
Inhibitory Effects of Various Compounds on Uptake by Mice Kidney Slices and Transporter-expressing HEK293 Cells. (**A**) Uptake of [^3^H]ergothioneine (1.0 μM) into mice kidney slices was performed in the presence or the absence of the indicated compounds at pH 7.4 and 37 °C for 3 min. Each bar represents the mean ± S.E.M. (n = 3 or 4) after subtraction of the uptake of [^3^H]ergothioneine in the absence of Na^+^. (**B**) Uptake of [^3^H]ergothioneine (1.0 μM) into Octn1-expressing HEK293 cells was performed in the presence or the absence of the indicated compounds at pH 7.4 and 37 °C for 15 sec. Each bar represents the mean ± S.E.M. (n = 3) after subtraction of the uptake of [^3^H]ergothioneine in the mock cells. (**C**) Effect of pH or Pept inhibitors on [^3^H]Gly-Sar uptake in mice kidney slices was performed at pH 7.4 or 6.0, and 37 °C for 5 min. Each bar represents the mean ± S.E.M. (n = 3). (**D**) Effect of dapagliflozin combination with D-galactose (30 mM) on [^14^C]αMG uptake into kidney mice slices was performed at pH 7.4 and 37 °C for 5 min. Each bar represents the mean ± S.E.M. (n = 3) after subtraction of the uptake of [^14^C]αMG in the absence of Na^+^. (**E**) Effect of dapagliflozin combination with D-glucose (0, 1 and 10 mM) on [^14^C]αMG uptake was performed at pH 7.4 and 37 °C for 5 min. Each bar represents the mean ± S.E.M. (n = 3) after subtraction of the uptake of [^14^C]αMG in the absence of Na^+^. * indicates a significant difference from the control (p < 0.05) by dunnett test.

In order to characterize the transporter-mediated uptake by kidney slices, effects of inhibitors for each transporter were examined. Cimetidine and carnitine, Octn1 inhibitors, significantly reduced Na^+^-dependent uptake of [^3^H]ergothioneine into mice kidney slices (Fig. 3A). The tendency of inhibition by cimetidine and carnitine was in accordance with Octn1-mediated uptake of [^3^H]ergothioneine into Octn1-expressing HEK293 cells (Fig. 3B). Moreover, the uptake of [^3^H]Gly-Sar at pH 7.4 was comparable with that at pH 6.0, and was decreased by 100 μM cephadroxil, a specific inhibitor of Pept2, and Gly-Leu (Fig. 3C). [^14^C]αMG uptake by kidney slices was decreased by 30 mM galactose, a specific inhibitor for Sglt1 (Fig. 3D). On the other hand, [^14^C]αMG uptake was not affected by 50 nM dapagliflozin, an Sglt2 selective inhibitor. Interestingly, in the presence of 50 nM dapagliflozin, the uptake of [^14^C]αMG decreased when 30 mM galactose or 1 mM D-glucose was simultaneously added (Fig. 3E). This observation is explained that both of Sglt1 and Sglt2 are functional in this method, but high affinity transporter Sglt1 is not functional in the presence of 30 mM of galactose or 1 mM of D-glucose, resulting in the clear observation of dapagliflozin effect on the apparent uptake of [^14^C]αMG in the presence of galactose or D-glucose

### Kinetics of Uptakes by Mice Kidney Slices and Transporter-expressing Cells

**Figure 4:**
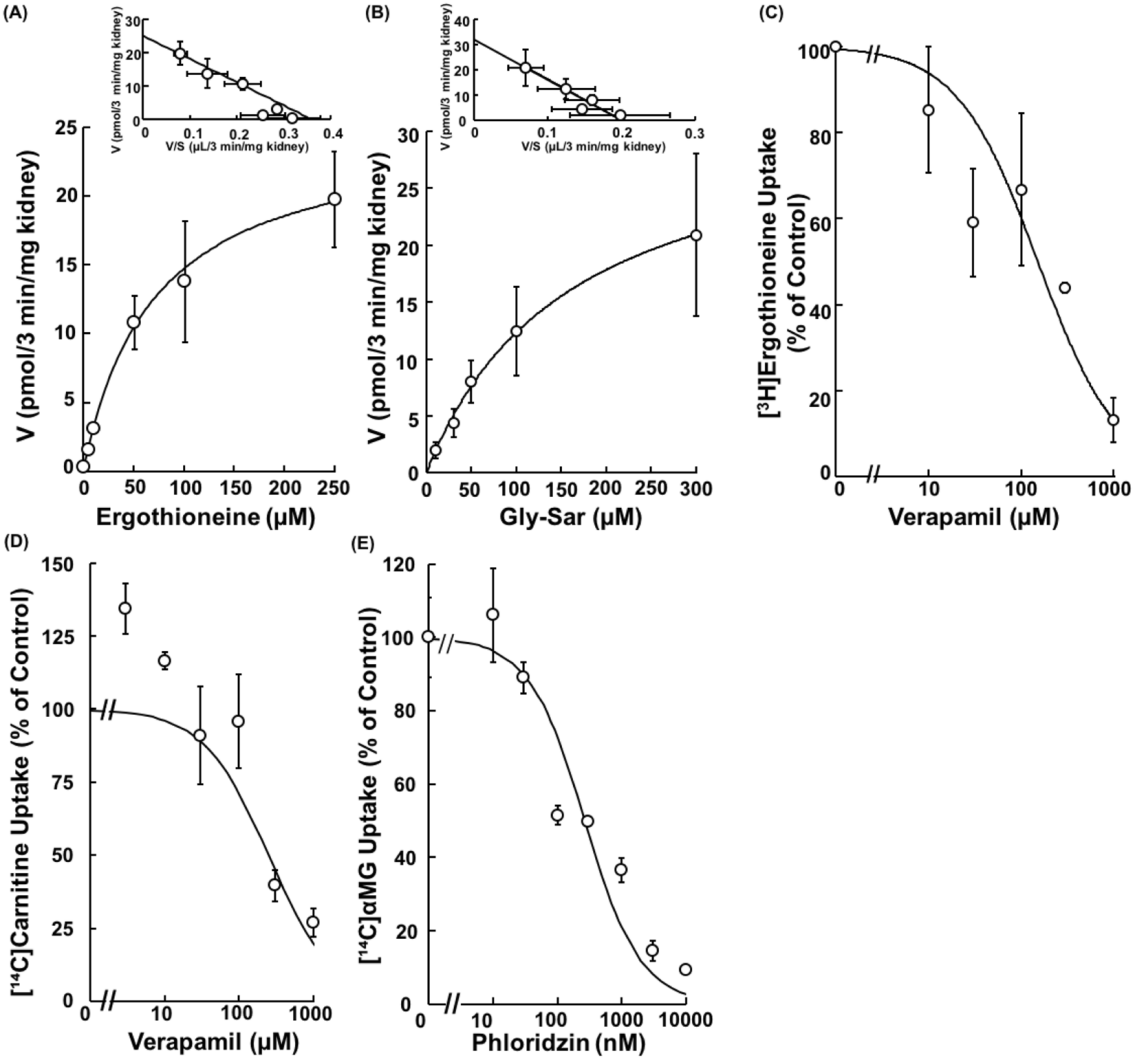
Concentration Dependences of Uptake by Mice Kidney Slices. (**A**) Uptake of [^3^H]Ergothioneine (1, 5, 10, 50, 100 and 250 μM) into mice kidney slices was measured at pH 7.4 and 37 °C for 3 min. The data were fitted to the Michaelis-Menten equation and Eadie-Hofstee plot by a nonlinear least-squares regression analysis. Each point represents the mean ± S.E.M. (n = 3 or 4), after subtraction of the uptake of [^3^H]Ergothioneine (1,000 μM). (**B**) Uptake of [^3^H]Gly-Sar (10, 30, 50, 100, 300 μM) into mice kidney slices was measured at pH 7.4 and 37 °C for 5 min. The data were fitted to the Michaelis-Menten equation and Eadie-Hofstee plot by a nonlinear least-squares regression analysis. Each point represents the mean ± S.E.M. (n = 3 or 4), after subtraction of the uptake of [^3^H]Gly-Sar (10 mM). (**C**) Uptake of [^3^H]ergothioneine (1.0 μM) into mice kidney slices was performed at pH 7.4 and 37 °C for 3 min in the absence or presence of verapamil (10, 30, 100, 300 and 1,000 μM). Each point represents the mean ± S.E.M. (n = 3), after subtraction of the uptake of [^3^H]ergothioneine in the absence of Na^+^. (**D**) Uptake of [^3^H]carnitine (1.0 μM) into mice kidney slices was performed at pH 7.4 and 37 °C for 3 min in the absence or the presence of verapamil (10, 30, 100, 300 and 1,000 μM). Each point represents the mean ± S.E.M. (n = 3), after subtraction of the uptake of [^3^H]carnitine in the absence of Na^+^. (**E**) Uptake of [^14^C]αMG (1.0 μM) into mice kidney slices was performed at pH 7.4 and 37 °C for 3 min in the absence or presence of phloridzin (10, 30, 100, 300, 1,000, 3,000 and 10,000 nM). Each point represents the mean ± S.E.M. (n = 3), after subtraction of the uptake of [^14^C]αMG in the absence of Na^+^.

*K*_*m*_ values of respective probe substrates and *IC*_50_ values of respective inhibitors were estimated (Fig. 4) in order to further confirm the transporter functions at the apical membrane of mouse kidney slices, and are summarized in Table 1. Concentration dependent uptakes of [^3^H]ergothioneine and [^3^H]Gly-Sar into mice kidney slices were monophasic in Eadie-Hofstee plots with the *K*_*m*_ values of 69.8 ± 27.6 µM and 161 ± 17 µM, respectively (Fig. 4A and B). The *IC*_50_ values of verapamil for ergothioneine uptake were 151 ± 46 µM and 271 ± 44 µM in mice kidney slices and Octn1-expressing HEK293 cells, respectively (Fig. 4C and Supplemental Fig. 2A). Moreover, the *IC*_50_ values of verapamil for carnitine uptake were 363 ± 142 µM and 94.1 ± 8.9 µM in mice kidney slices and Octn2-expressing HEK293 cells, respectively (Fig. 4D and Supplemental Fig. 2B). The *IC*_50_ values of phloridzin for [^14^C]αMG uptake was 0.265 ± 0.084 nM in mice kidney slices (Fig. 4E). The *K*_*m*_ and *IC*_50_ values for substrates and inhibitors of each transporter were mostly comparable to those obtained in transporter-transfected cell models (Table 1).

**Table 1:** Summary of the calculated *K*_*m*_ and *IC*_50_ values.

Transporter	Compound	Mice Kidney Slice (µM)	Expression Model (µM)
Octn1	Ergothioneine (*K*_*m*_)	69.8 ± 27.6	4.68^22^
	Verapamil (*IC*_50_)	151 ± 46	271 ± 44
Octn2	Verapamil (*IC*_50_)	363 ± 142	94.1 ± 8.9
Sglt1/2	Phloridzin (*IC*_50_)	0.265 ± 0.084	0.299 (Sglt1)^33^
			0.0726 (Sglt2)^33^
Pept1/2	Gly-Sar (*K*_*m*_)	161 ± 17	600 (PEPT1)^37^
			79.5 (PEPT2)^31^

## Discussion

In the present study, we evaluated a usefulness of kidney slices for the study of renal tubular apical membrane transporters as an additional *in vitro* experimental model which does not require cumbersome preparation procedures. Although kidney slices have been widely used for the renal tubular basolateral membrane transport, it has been unclear whether the activity of apical membrane specific transports is functional and detectable in prepared kidney slices or not. Among transporters expressed at apical membranes of proximal tubular epithelial cells, four types of transporters, including Octn1, Octn2, Sglt1/2, and Pept1/2, were selected in the present study, since probe substrates, ergothioneine, carnitine, αMG and Gly-Sar, respectively, can discriminate transports between apical and basolateral membranes, due to its Na^+^-dependence and/or specific expression at the apical membrane. Then, transport of probe substrates was measured in the presence and in the absence of driving force or inhibitors of each transporter for apical uptake (Fig. 1A–D). As a result, we clearly observed Na^+^-depedent uptake of ergothioneine, carnitine, and αMG, and reduction of Gly-Sar uptake in the presence of Glu-Leu, suggesting detection of apical uptake transporters Octn1, Octn2, Sglt1/2, and Pept1/2. On the other hand, Wedeen and Weiner reported that alpha-aminoisobutyric acid may not be taken up into renal cells from luminal side, because luminal staining of radio-labeled compound was not detected. The reason why Wedeen and Weiner failed to observe apical uptake transporters might be due to low sensitivity to detect signal at luminal side by their method, which partly come from sensitivity of radio-autography and thicker kidney tissue slices than those in the present study.

As shown in Fig. 3A and B, Octn1 inhibitor, carnitine and cimetidine reduced [^3^H]ergothioneine uptake by kidney slices with mostly correspondence features to Octn1-transfected cells, in spite of the presence of other cation transporters such as Octn2, Oct2 and Mate1 in kidney slices. Considering that *K*_*m*_ value of carnitine for Octn2 is 22.1 µM^19^ and cimetidine inhibits Oct2 and Mate1 with *IC*_50_ values of 8.0 and 16.3 µM^27,28^, respectively, no significant inhibition by 0.5 mM carnitine and 0.1 mM cimetidine suggest that uptake of [^3^H]ergothioneine by kidney slices was not explained by Octn2, oct2 or Mate1. Furthermore, unknown transporters other than Octn1 might be involved in the Na^+^-dependent uptake of ergothioneine. Therefore, Octn1 specificity was confirmed using kidney slices obtained from Octn1^−/−^ mice. Under condition where no changes were observed in [^14^C]αMG uptake in kidney slices from Octn1^−/−^ mice (Fig. 2B), specific decrease in ergothioneine uptake by kidney slices from Octn1^−/−^ mice confirmed that ergothioneine transport is ascribed to Octn1.

To characterize the transport activity of Pept1/2, the uptake of [^3^H]Gly-Sar was characterized. As shown in Fig. 3C, the uptakes of [^3^H]Gly-Sar were comparable at pH 7.4 and 6.0. This result was in accordance with reported characteristics that uptake of [^3^H]Gly-Sar by Pept2 was similar at pH 7.4 and 6.0, whereas the uptake by Pept1 at pH 6.0 was higher than that at pH 7.4^29^. Furthermore, the uptake was reduced by 100 μM cephadroxil, which is a selective concentration only to Pept2, to the same extent as Gly-Leu, which is an inhibitor for both Pept1 and Pept2. This observation is explained by major contribution of Pept2 in Gly-Sar uptake by the kidney slices^29^, along with abundant expression of Pept2 compared with Pept1 in mice kidney^30^. Moreover, Eadie-Hofstee plot of concentration-dependent uptake of Gly-Sar was apparently monophasic with the *K*_*m*_ value of 161 ± 17 µM (Fig. 4B), which is comparable to reported *K*_*m*_ value of 79.5 µM for [^3^H]Gly-Sar uptake by Pept2^31^. The present observation indicated that Gly-Sar uptake into kidney slices was mainly mediated by Pept2.

Although αMG uptake exhibited clear Na^+^ dependence, which can be explained by Sglt transporters, discrimination of Sglt1 and Sglt2 was not clear. However, the difference in affinity to D-glucose between Sglt1 and Sglt2 could be used for discrimination between those two glucose transporters. In kidney, Sglt2 is expressed at apical membrane in early proximal tubule and Sglt1 is at those in distal part of the proximal tubule. Therefore, physiologically tubular D-glucose uptake across the apical membrane occurs in the early proximal tubule by low-affinity/high-capacity Sglt2, and then remaining D-glucose are reabsorbed by the high-affinity/low-capacity Sglt1 in further distal parts of the proximal tubule^32^. However, in the present kidney slices, Sglt1 and Sglt2 could be active, since the prepared slices contains both of cortex and medulla part of kidney. In order to confirm both Sglt1/2 are active in the prepared slices, we conducted inhibition study using selective Sglt2 inhibitor dapagliflozin at three different D-glucose concentrations (0, 1 and 10 mM). Uptake of αMG was not reduced by Sglt2 inhibitor dapagliflozin (*IC*_50_ values: 5.12 nM^33^) at D-glucose free condition. Whereas dapagliflozin inhibited αMG uptake in the presence of 1 mM D-glucose, which is physiologically relevant concentration in the tubular lumen and Sglt1 is saturated, or 30 mM D-galactose, which is a selective Sglt1-inhibiting condition (Fig. 3D and E). Furthermore, in the presence of 10 mM D-glucose, which is a condition that both of Sglt1 and Sglt2 are not functional due to saturation, no further decrease by dapagliflozin was observed (Fig. 3E). These results demonstrated that Sglt1/2 take up αMG in parallel into kidney slice, which explains apparent no change in αMG uptake by Sglt2 inhibitor alone in this method.

In Fig. 1B, [^3^H]carnitine was slightly accumulated into the kidney slices in the absence of Na^+^, suggesting Na^+^-independent carrier mediated transport taking into consideration water-soluble property of carnitine. It was reported that Na^+^-independent uptake of carnitine by brush-border membrane vesicles (BBMVs) of Octn2-dificient juvenile visceral steatosis (jvs) is present^34^. The responsible transporter molecule has not been identified yet; however, we reported carnitine is transported by OCTN3 in a Na^+^-independent manner^19^. In addition, unidentified transporters might be involved in the Na^+^-independent uptake of carnitine in our mouse kidney slices.

As shown in Fig. 4 and Table 1, the *K*_*m*_ value of 81.5 μM for ergothioneine transport into kidney slices was 17-times higher than that of 4.68 μM into Octn1-expressing HEK293 cells in the previous report^22^. However, functional Octn1 was clearly demonstrated by comparison of the uptakes by kidney slices from wild-type and Octn1^−/−^ mice (Fig. 2A), indicating that this discrepancy cannot be explained by contribution of multiple transporters in kidney slices. Potential explanations are 1) difference in nominal and actual ergothioneine concentrations around Octn1 protein molecules in kidney slices and recombinant system and 2) difference in post-translational modifications of Octn1^16^ between native Octn1 in kidney tissues and transporter-gene transfected cells.

For the evaluation of apical transporters, BBMVs have been extensively and widely applied. Merits of kidney slices compared with BBMVs considered to be as follows; 1) When metabolic enzymes such as UDP-glucuronosyltransferase (UGT) and carboxylesterase (CES) in kidney are involved, both of transport and metabolic processes can be simultaneously evaluated. 2) Transporters with unknown characteristics such as driving force can be evaluated in kidney slice, whereas uptake study by membrane vesicles is fully dependent on the uptake medium conditions. 3) In membrane vesicles, adsorption of lipophilic compound to filter paper often disturbs measurement of uptake, whereas no filter paper is used in kidney slice method. 4) Although BBMVs include both of right-side out and inside-out vesicles during uptake measurement, kidney slices can maintain physiologically directional transport. On the other hand, there are several disadvantages in kidney slice method compared with BBMVs as followings; 1) kidney slices cannot be stored frozen. 2) It might be difficult to evaluate transport of compound that is extensively metabolized inside of the cells. 3) It is difficult to distinguish between apical and basolateral transports in case that similar transporters are contributing both at apical and basolateral membranes and differentiation of those transporter by using different driving force, selective inhibitor or any other ways is not possible. 4) At this moment, it is unclear whether ATP-dependent transports could be evaluated or not and further studies are required.

In conclusion, it was clearly demonstrated that kidney slices are useful *in vitro* model to evaluate apical uptake transporters, Octn1, Octn2, Sglt1/2 and Pept1/2 in renal tubular cells. Based on these results, it is expected that other transporters at apical membrane can be evaluated by this method. Accordingly, this model is a simple and promising tool for an evaluation of renal disposition of drugs and nutrients by enabling analysis of both the apical and basolateral transports by controlling the experimental conditions.

## Methods

### Materials

[^3^H]Ergothioneine (3.7 GBq/mmol, custom-made) and [^14^C]αMG, (39.3 mCi/mmol), [^3^H]Gly-Sar (2.8 Ci/mmol) were purchased form Moravek Biochemicals (Brea, CA, USA). L-[Methyl-^3^H]-carnitine (80 Ci/mmol) and [carboxyl-^14^C]inulin carboxyl (2.5 mCi/g) were purchased from American Radiolabeled Chemicals Inc. (St. Louis, MO). All other chemicals and reagents were commercial products of reagent grade.

### Animals

Male ICR mice (40 ± 5 g body weight) were purchased from Japan SLC (Hamamatsu, Japan). Octn1^−/−^ and wild-type littermates were generated as described previously^22^, and were backcrossed to C57BL/6 J strain. Mice were housed four per cage with free access to commercial chow and tap water, and were maintained on a 12 h dark/light cycle in an air-controlled room (temperature, 24.0 ± 1 °C; humidity, 55 ± 5%). All animal studies were approved by the Kanazawa University Institutional Animal Care and Use Committee (Permit number, AP-163750), and the all animal experiments in this study were performed in accordance with the committee.

### Uptake Studies using Mice Kidney Slices

Uptake studies using mouse kidney slices were carried out as described in a previous report after slight modifications^13^. The slices of whole kidneys were prepared with a microslicer (Zero 1; Dosaka EM, Kyoto, Japan), and the renal pelvis portion was removed. Prepared slices (0.3 mm thick) of whole kidneys from male mice were immediately put in ice-cold oxygenated transport buffer (130 mM NaCl, 4.8 mM KCl, 1.2 mM CaCl_2_, 1.2 mM MgSO_4_, 1.2 mM KH_2_PO_4_ and 25 mM HEPES, adjusted to pH 7.4). When uptake was conducted in Na^+^-free condition, NaCl was replaced with N-methyl-D-glucamine (NMG) in transport buffer. Three or four slices from one mouse, weighing each slice 2 to 10 mg, were randomly selected and then pre-incubated in a 24-well plate with 1.0 ml of oxygenated transport buffer in each well at 37 °C for 5 min. After pre-incubation, the kidney slices were put into transport buffer containing radiolabeled substrates with or without inhibitors to initiate the uptake reaction. The uptake reactions were carried out at 37 °C for an appropriate time, and then each slice was rapidly removed from the transport buffer, washed twice in ice-cold transport buffer, blotted on filter paper and weighed. For measurement of radiolabeled compounds, the slices were dissolved in 0.15 ml of 1 N NaOH. Then, the lysate was neutralized with HCl. The apparent uptake of [^14^C]inulin was evaluated in independent experiments (n = 3) to estimate the volume of water adhering to the kidney slices after an incubation. In this study, observed extracellular adhered water space was 0.128 ± 0.004 μL/5 min/mg kidney. Viability of the isolated kidney slices was assessed as described in the supplemental file.

### Uptake Studies Using Transporter-expressing Cells

Mouse Octn1- and Octn2-expressing and empty vector transfected (mock) HEK293 cells were established in our previous study^19^. The cells were grown in Dulbecco’s modified Eagle’s medium (DMEM) containing 10% (v/v) fetal bovine serum (FBS) (Life Technologies, Carlsbad, CA, USA), 100 units/ml penicillin and 100 mg/ml streptomycin at 37 °C in an atmosphere of 5% CO_2_. Octn1- and Octn2-expressing HEK293 cells and mock cells were plated onto 24-well poly-L-lysine-coated tissue culture plates at a density of 1.0 × 10^5^ cells/well. The cells were cultured for 2 days, and then pre-incubated with 0.5 ml of transport buffer (125 mM NaCl, 4.8 mM KCl, 5.6 mM D-glucose, 1.2 mM CaCl_2_, 1.2 mM KH_2_PO_4_, 1.2 mM MgSO_4_ and 25 mM HEPES to adjusted to pH 7.4) in each well at 37 °C for 15 min. Uptake reaction was initiated by adding transport buffer containing a radiolabeled substrate with or without inhibitors to each well, and incubation was carried out at 37 °C for a certain period of time. The uptake was terminated by adding ice-cold transport buffer. Then, the cells were washed three times with the transport buffer to remove adhering substrate, and lysed with 0.25 mL of 0.01% Triton X. The cellular protein content was measured with a protein assay kit (Bio-Rad) using bovine serum albumin (Fraction V, Sigma Aldrich, St. Louis, MO) as a standard^35^.

### Data Analysis

Radioactivity was quantified with a liquid scintillation counter (Aloka, Tokyo, Japan). To calculate kinetic parameters for carrier-mediated uptake of test substrates, uptake rate was fitted to eq. (1) by means of a nonlinear least-squares regression analysis using MULTI program^36^:

1 $$v={V}_{max}\times s/({K}_{m}+s)$$

where v, s, *K*_*m*_, and *V*_*max*_ are the uptake rate of substrate, the substrate concentration in the medium, the apparent Michaelis-Menten constant, and the maximal uptake rate, respectively.

The half-inhibitory concentration (*IC*_50_) values of inhibitors were obtained by examining their inhibitory effects on uptake of test substrates according to the following equation (2):

2 $$ \% \,{\rm{of}}\,{\rm{control}}=I{C}_{50}/(I{C}_{50}+I)$$

where % of control represents inhibition rate in the presence of inhibitors and I is inhibitor concentration.

## Electronic supplementary material

Kidney Slice Supplemental document
