# Supplementary material for: Usefulness of kidney slices for functional analysis of apical reabsorptive transporters
Source: Sci Rep. 2017 Oct 9;7:12814. doi: 10.1038/s41598-017-12828-z (PMC5634478; doi:10.1038/s41598-017-12828-z)
Supplement: Supplementary file 1 — Kidney Slice Supplemental document [file 41598_2017_12828_MOESM1_ESM.doc]

**SUPPLEMENTARY INFORMATION**

Title: Usefulness of kidney slices for functional analysis of apical reabsorptive transporters

Hiroshi Arakawa^1^, Ikumi Washio^2^, Natsumi Matsuoka^1^, Hikaru Kubo^1^, Angelina Yukiko Staub^1^, Noritaka Nakamichi^3^, Naoki Ishiguro^2^, Yukio Kato^3^, Takeo Nakanishi^1^, Ikumi Tamai^1,*^

^1^Department of Membrane Transport of Biopharmaceutics, Faculty of Pharmaceutical Sciences, Institute of Medical, Pharmaceutical and Health Sciences, Kanazawa University, Kakuma-machi, Kanazawa 920-1192, Japan

^2^Pharmacokinetics and Non-Clinical Safety Department, Nippon Boehringer Ingelheim Co., Ltd., Kobe, Japan.

^3^Laboratory of Molecular Pharmacotherapeutics, Faculty of Pharmaceutical Sciences, Institute of Medical, Pharmaceutical and Health Sciences, Kanazawa University, Kakuma-machi, Kanazawa 920-1192, Japan

* To whom corresponding to

Department of Membrane Transport and Biopharmaceutics, Faculty of Pharmaceutical Sciences, Kakuma-machi, Kanazawa, Ishikawa, 920-1192, Japan,

Tel: +81-76-234-4479, Fax: +81-76-264-6284,

E-mail: tamai@p.kanazawa-u.ac.jp

**Supplemental Materials**

**Method**

**Measurement of ATP content in kidney slices**

Mouse kidney slices in the were incubated for 0.25, 1, 2, 3, 4 and 6 hr at pH 7.4 and 37℃ in the presence or the absence of Na^+^. The kidney slices were collected, and was immediately frozen and stored in liquid nitrogen for subsequent ATP analysis. The tissues were homogenized by bullet blender, and centrifuged at 1,000 g for 10 min at 4°C. The supernatant was collected for analyzing ATP using “Tissueno” ATP assay kit (TOYO B-Net Co., LTD, Tokyo, Japan). Relative ATP content was calculated according to amount of luminescence versus the ATP standard curve.

**Supplemental Figure**

**Supplemental Figure 1. Effect of Pre-Incubation Time on Uptake of [^3^H]Ergothioneine , [^3^H]Carnitine, [^14^C]αMG and [^3^H]Gly-Sar and ATP Contents by Mouse Kidney Slices**

Uptake of (A) [^3^H]ergothioneine (1.0 μM), (B) [^3^H]carnitine (1.25 nM) and (C)[^14^C]αMG (2.54 µM) were performed by mouse kidney slices for 5 min at pH 7.4 and 37°C for 5 min in the presence or in the absence of Na^+^ after pre-incubation for 0.25, 1, 2, 3, 4 and 6 hr at pH 7.4 and 37°C. Closed and open circles represent each substrate uptake in the presence or the absence of Na^+^, respectively. Uptake of (D)[^3^H]Gly-Sar (35.7 nM) was performed by mouse kidney slices for 5 min at pH 7.4 and 37°C for 5 min in the presence or in the absence of Gly-Leu (5.0 mM) after pre-incubation for 0.25, 1, 2, 3, 4 and 6 hr at pH 7.4 and 37℃. Closed and open circles represent each substrate uptake in the absence or the presence of Gly-Leu, respectively. Cellular ATP content of mouse kidney slices in the presence (E) or absence (F) of Na^+^ was measured after pre-incubation at pH 7.4 and 37℃ for 0.25, 1, 2, 3, 4 and 6 hr. Closed and open circles represent ATP content in the presence or absence of Na^+^. Each result represents the mean ± S.E.M. (n=3). * indicates a significant difference from the uptake after pre-incubation for 0.25 hr (p＜0.05) by Student’s t-test.

**Supplemental Figure 2.** **Concentration Dependences of Uptake by Transporter-expressing Cells.** (A) Uptake of [^3^H]ergothioneine (1.0 μM) by Octn1-expressing HEK293 cells was performed at pH 7.4 and 37°C for 15 sec in the absence or the presence of verapamil (3, 10, 30, 100, 300 and 1,000 μM). Each point represents the mean ± S.E.M. (n = 3 or 4), after subtraction of the uptake of [^3^H]ergothioneine in mock cells. (B) Uptake of [^3^H]carnitine (1.0 μM) by Octn2-expressing HEK293 cells was performed at pH 7.4 and 37°C for 15 sec in the absence or presence of verapamil (3, 10, 30, 100, 300 and 1000 μM). Each point represents the mean ± S.E.M. (n = 3 or 4), after subtraction of the uptake of [^3^H]carnitine in mock cells.
